# Efficacy and safety of canagliflozin compared with placebo in older patients with type 2 diabetes mellitus: a pooled analysis of clinical studies

**DOI:** 10.1186/1472-6823-14-37

**Published:** 2014-04-18

**Authors:** Alan Sinclair, Bruce Bode, Stewart Harris, Ujjwala Vijapurkar, Cristiana Mayer, Albert Fung, Wayne Shaw, Keith Usiskin, Mehul Desai, Gary Meininger

**Affiliations:** 1 Luton & Dunstable University Hospital; Bedfordshire and Hertfordshire Postgraduate Medical School, University of Bedfordshire, Putteridge Bury Campus, Hitchin Road, Luton LU2 8LE, UK; 2 Atlanta Diabetes Associates, 77 Collier Rd. Suite 2080, Atlanta, GA 30309, USA; 3 University of Western Ontario, 245-100 Collip Circle, UWO Research Park, London, Ontario N6G-4X8, Canada; 4 Janssen Research & Development, LLC, 920 Route 202 South, Raritan NJ 08869, USA; 5 Janssen Research & Development, LLC, 1125 Trenton-Harbourton Road, Titusville NJ 08560, USA

**Keywords:** Canagliflozin, Type 2 diabetes mellitus, Sodium glucose co-transporter 2 (SGLT2) inhibitor, Antihyperglycaemic agent, Older patients

## Abstract

**Background:**

Canagliflozin is a sodium glucose co-transporter 2 inhibitor developed for the treatment of patients with type 2 diabetes mellitus (T2DM). The efficacy and safety of canagliflozin were evaluated in patients with T2DM <65 and ≥65 years of age.

**Methods:**

Pooled data from 4 randomised, placebo-controlled, 26-week, Phase 3 studies (N = 2,313) evaluating canagliflozin 100 and 300 mg were analysed by age: <65 years (n = 1,868; mean age, 52.8 years) or ≥65 years (n = 445; mean age, 69.3 years). Efficacy evaluations included change from baseline in glycaemic parameters and systolic blood pressure (BP), and percent change from baseline in body weight. Assessment of safety/tolerability included adverse event (AE) reports, incidence of documented hypoglycaemia, and percent change from baseline in fasting plasma lipids.

**Results:**

Canagliflozin 100 and 300 mg reduced HbA_1c_ and fasting plasma glucose relative to placebo in patients <65 and ≥65 years of age. Both canagliflozin doses reduced body weight and systolic BP relative to placebo in patients <65 and ≥65 years of age. Incidence of overall AEs was similar across all treatment groups in patients <65 and ≥65 years of age. Incidences of serious AEs and AE-related discontinuations were similar across all treatment groups in patients <65 years of age and higher with canagliflozin 100 mg than other groups in patients ≥65 years of age. As in patients <65 years of age, incidences of genital mycotic infections and osmotic diuresis-related AEs were higher with canagliflozin relative to placebo in those ≥65 years of age. Incidences of urinary tract infections (UTIs), renal-related AEs, AEs related to volume depletion, and documented hypoglycaemia episodes were similar across all treatment groups in patients ≥65 years of age; no notable trends were observed with canagliflozin 100 and 300 mg relative to placebo in these AEs among patients <65 years of age. Changes in lipid parameters with canagliflozin were similar in both age subsets.

**Conclusions:**

Canagliflozin improved glycaemic control, body weight, and systolic BP, and was generally well tolerated in older patients with T2DM.

**Trial registration:**

ClinicalTrials.gov, NCT01081834; NCT01106677; NCT01106625; NCT01106690.

## Background

Advancing age, the increased prevalence of medical comorbidities, and the emergence of frailty can impact the selection of antihyperglycaemic agent (AHA) therapies for treating older patients with type 2 diabetes mellitus (T2DM) [1,2]. Important safety considerations in this population include those related to renal or hepatic impairment, cardiovascular disease, polypharmacy, and self-management ability [1,3-5]. Hypoglycaemia is of particular concern for older patients with T2DM because the risk of experiencing a hypoglycaemia episode increases with advancing age [5,6]; hypoglycaemia episodes associated with AHA use are a major cause of hospitalisations among older patients with T2DM [7]. Thus, the benefit/risk profiles of available AHA therapies are important considerations in treating older patients with T2DM.

Canagliflozin is a sodium glucose co-transporter 2 (SGLT2) inhibitor developed for the treatment of patients with T2DM [8-16]. Canagliflozin lowers the renal threshold for glucose excretion (RT_G_) and increases urinary glucose excretion (UGE) in individuals with hyperglycaemia, resulting in decreased plasma glucose as well as an osmotic diuresis and net caloric loss [8,17,18]. In Phase 3 studies in patients with T2DM, canagliflozin has been shown to improve glycaemic control and reduce body weight and blood pressure (BP) as monotherapy or in combination with a variety of background AHAs [9-16].

In a Phase 3 study of older patients with T2DM ≥55 to ≤80 years of age, canagliflozin 100 and 300 mg significantly reduced HbA_1c_, fasting plasma glucose (FPG), body weight, and systolic BP relative to placebo, and were generally well tolerated [11]. Incidences of genital mycotic infections, urinary tract infections (UTIs), and osmotic diuresis-related adverse events (AEs) were higher with canagliflozin than placebo, consistent with findings from other studies of canagliflozin in generally younger patient populations [9,10,12,14-16]. To further evaluate the efficacy and safety of canagliflozin in older patients with T2DM, an analysis of pooled data from 4 randomised, placebo-controlled, Phase 3 studies (the aforementioned study in older patients was not included in this pooled analysis) was performed, with results reported for subsets of patients <65 and ≥65 years of age.

## Methods

### Study design and patient population

This pooled analysis evaluated canagliflozin 100 and 300 mg and placebo using data from subsets of patients with T2DM <65 and ≥65 years of age from 4 randomised, double-blind, placebo-controlled, Phase 3 studies. These studies each included a 26-week, double-blind, core treatment period and a 26-week extension period, and assessed canagliflozin as monotherapy [9] or added on to metformin [14], metformin plus sulphonylurea [15], and metformin plus pioglitazone (Table 1). Data from the 26-week core treatment periods of each study were included in this pooled analysis; the high glycaemic subset (HbA_1c_ >10.0% and ≤12.0%) of the monotherapy study, which was not placebo controlled, and the sitagliptin arm of the add-on to metformin study were excluded. For the pooled dataset, the mean duration of exposure to study drug was approximately 24 weeks in each treatment group.

**Table 1:** Summary of patient populations

		**Inclusion criteria**			**Patients contributing to pooled analysis, n**				
**Study**	Duration^*^	**Age, y**	**HbA**_**1c**_**, %**	**eGFR,**	**PBO**	**CANA**	**CANA**	**Total**	**Aged**
				**mL/min/1.73 m**^**2**^		**100 mg**	**300 mg**		**≥65 y**
Monotherapy	26 weeks	≥18 to ≤80	≥7.0 and ≤10.0	≥50	192	195	197	584	118
Add-on to MET	26 weeks	≥18 to ≤80	≥7.0 and ≤10.5	≥55	183	368	367	918	149
Add-on to MET + SU	26 weeks	≥18 to ≤80	≥7.0 and ≤10.5	≥55	156	157	156	469	85
Add-on to MET + PIO	26 weeks	≥18 to ≤80	≥7.0 and ≤10.5	≥55	115	113	114	342	93
**Overall total, n**					**646**	**833**	**834**	**2,313**	**445**

eGFR, estimated glomerular filtration rate; PBO, placebo; CANA, canagliflozin; MET, metformin; SU, sulphonylurea; PIO, pioglitazone.

*Assessment time point; mean treatment exposure of 24.2, 24.3, and 23.8 weeks with canagliflozin 100 and 300 mg and placebo, respectively.

Key inclusion criteria for the individual studies are summarised in Table 1. In general, eligible patients were those with T2DM ≥18 and ≤80 years of age, with HbA_1c_ ≥7.0% and ≤10.5% and estimated glomerular filtration rate (eGFR) ≥55 mL/min/1.73 m^2^ at screening. Key exclusion criteria that were common across studies included repeated FPG ≥15.0 mmol/L during the pretreatment phase; history of type 1 diabetes; history of cardiovascular (CV) disease (including myocardial infarction, unstable angina, revascularisation procedure, or cerebrovascular accident) within 3 months prior to screening; and alanine aminotransferase (ALT) level >2.0 times the upper limit of normal (ULN) or total bilirubin >1.5 the ULN at screening.

In each study, eligible patients who were on protocol-specified background diabetes treatment directly entered a 2-week, placebo run-in period; those not on protocol-specified background diabetes therapy entered an 8- to 12-week AHA adjustment/dose stabilisation period prior to the run-in period. Patients were to remain on their stable diabetes treatment regimen through the end of the double-blind treatment period. Randomisation to treatment group (canagliflozin 100 or 300 mg or placebo) was stratified to ensure adequate distribution of specific patient characteristics (eg, whether a patient entered the AHA adjustment/dose stabilisation period) across treatment groups. After randomisation, HbA_1c_ and FPG were masked to study centres unless pre-defined criteria for initiation of glycaemic rescue therapy based on HbA_1c_ or FPG values were met. Study databases were locked at the primary assessment time point (Week 26) and studies were unblinded by the sponsor for regulatory filing. Blinding was maintained for patients and study centre and local sponsor personnel throughout the double-blind treatment period.

Glycaemic rescue therapy was initiated during the double-blind treatment period for patients who met pre-specified criteria (in general, FPG >15.0 mmol/L after Day 1 to Week 6, >13.3 mmol/L after Week 6 to Week 12, and >11.1 mmol/L after Week 12 to Week 26). The agent for rescue therapy in each study was selected to be complementary to the protocol-specified background AHA therapy.

The studies were conducted in accordance with the ethical principles that comply with the Declaration of Helsinki, and are consistent with Good Clinical Practices and applicable regulatory requirements. Approval was obtained from institutional review boards and independent ethics committees for participating centres (Additional file 1), and patients gave written informed consent prior to participation.

### Endpoints and assessments

Efficacy endpoints evaluated at Week 26 included change from baseline in HbA_1c_, FPG, and systolic and diastolic BP, and percent change from baseline in body weight (reported for prior to rescue medication).

Assessments of overall safety and tolerability were based on AEs, safety laboratory tests, 12-lead electrocardiograms, vital sign measurements, physical examinations, and self-monitored blood glucose. The incidence of selected AEs, including UTIs, genital mycotic infections, AEs related to osmotic diuresis and volume depletion, and renal-related AEs, were also evaluated. Documented hypoglycaemia episodes included biochemically confirmed episodes (concurrent fingerstick or plasma glucose ≤3.9 mmol/L, with or without symptoms) and severe episodes (ie, those requiring the assistance of another individual or resulting in seizure or loss of consciousness).

Percent changes from baseline in fasting plasma lipids, including triglycerides, high-density lipoprotein cholesterol (HDL-C), low-density lipoprotein cholesterol (LDL-C), LDL-C/HDL-C ratio, and non–HDL-C, were assessed as safety parameters.

### Statistical analyses

Efficacy endpoints were analysed in the modified intent-to-treat (mITT) population consisting of randomised patients who received ≥1 dose of study drug. The last observation carried forward (LOCF) approach was used to impute missing efficacy data. For patients who received rescue therapy, the last post-baseline value prior to initiation of rescue therapy was used for efficacy analyses. Changes from baseline in efficacy parameters at Week 26 were assessed using an analysis of covariance (ANCOVA) model including treatment and study as fixed effects and baseline values as covariates. Least squares (LS) means and 2-sided 95% confidence intervals (CIs) were estimated for the comparisons of each canagliflozin dose versus placebo. Fasting plasma lipid parameters were assessed in the safety analysis set (identical to the mITT population) using a similar ANCOVA model as for efficacy endpoints. Statistical testing of comparisons of canagliflozin versus placebo within each age group, and of comparisons between age groups, was not conducted (not pre-specified). Therefore, no *P* values are reported; however, 95% CIs are provided.

## Results

### Patient disposition and baseline characteristics

Of the patients in the pooled population who were randomised and dosed, 85.9% and 85.4% of those <65 and ≥65 years of age, respectively, completed the 26-week treatment period. A higher proportion of patients in the placebo group compared with the combined canagliflozin group discontinued before the Week 26 visit among patients <65 years of age (18.2% vs 12.6%) and those ≥65 years of age (17.5% vs 13.3%). Baseline demographic and disease characteristics were generally similar across treatment groups within each age subset (Table 2). Patients ≥65 years of age had a lower mean baseline eGFR, a longer mean duration of T2DM, and a higher proportion with cardiac disorders and on antihypertensive medication compared with the <65 years subset.

**Table 2:** Baseline demographic and disease characteristics^*^

	**Patients <65 y**			**Patients ≥65 y**		
**Characteristic**	**PBO**	**CANA 100 mg**	**CANA 300 mg**	**PBO**	**CANA 100 mg**	**CANA 300 mg**
	**(n = 509)**	**(n = 674)**	**(n = 685)**	**(n = 137)**	**(n = 159)**	**(n = 149)**
Sex, n (%)						
Male	263 (51.7)	327 (48.5)	327 (47.7)	71 (51.8)	81 (50.9)	77 (51.7)
Female	246 (48.3)	347 (51.5)	358 (52.3)	66 (48.2)	78 (49.1)	72 (48.3)
Age, y	53.0 ± 8.1	52.6 ± 8.1	52.8 ± 7.8	68.7 ± 3.5	70.0 ± 3.6	69.1 ± 3.5
Race, n (%)^†^						
White	358 (70.3)	455 (67.5)	482 (70.4)	112 (81.8)	136 (85.5)	128 (85.9)
Black or African American	23 (4.5)	40 (5.9)	43 (6.3)	5 (3.6)	3 (1.9)	5 (3.4)
Asian	75 (14.7)	99 (14.7)	92 (13.4)	7 (5.1)	4 (2.5)	8 (5.4)
Other^‡^	53 (10.4)	80 (11.9)	68 (9.9)	13 (9.5)	16 (10.1)	8 (5.4)
HbA_1c_, %	8.1 ± 1.0	8.0 ± 0.9	8.0 ± 1.0	7.8 ± 0.8	7.9 ± 0.9	7.9 ± 0.9
FPG, mmol/L	9.3 ± 2.2	9.4 ± 2.3	9.4 ± 2.3	9.0 ± 2.1	9.6 ± 2.4	9.6 ± 2.6
Body weight, kg	90.1 ± 22.3	91.0 ± 22.7	89.3 ± 22.8	86.3 ± 19.4	84.6 ± 19.7	84.6 ± 17.7
BMI, kg/m^2^	32.2 ± 6.7	32.7 ± 6.6	32.3 ± 6.7	30.8 ± 5.0	30.8 ± 5.2	30.5 ± 5.2
Systolic BP, mmHg	127.4 ± 13.2	126.9 ± 12.6	127.6 ± 12.6	132.7 ± 13.1	132.5 ± 12.8	134.3 ± 12.5
eGFR, mL/min/1.73 m^2^	90.0 ± 19.9	90.9 ± 19.0	91.3 ± 18.9	75.9 ± 15.1	77.3 ± 14.6	77.4 ± 13.5
Duration of diabetes, y	6.7 ± 5.4	6.4 ± 5.2	6.7 ± 5.4	10.2 ± 7.9	10.5 ± 7.0	10.8 ± 8.1
Patients with cardiac disorders, n (%)^§^	64 (12.6)	85 (12.6)	87 (12.7)	39 (28.5)	46 (28.9)	45 (30.2)
Patients on antihypertensive medications, n (%)^||^	305 (59.9)	415 (61.6)	408 (59.6)	110 (80.3)	120 (75.5)	115 (77.2)

PBO, placebo; CANA, canagliflozin; FPG, fasting plasma glucose; BMI, body mass index; BP, blood pressure; eGFR, estimated glomerular filtration rate; SD, standard deviation; MedDRA, Medical Dictionary for Regulatory Activities.

^*^Data are mean ± SD unless otherwise indicated.

^†^Percentages may not total 100.0% due to rounding.

^‡^Includes American Indian or Alaska Native, Native Hawaiian or other Pacific Islander, multiple, other, unknown, and not reported.

^§^Defined based on the MedDRA v13.1 system organ class for cardiac disorders from medical history.

^||^Includes agents acting on the renin-angiotensin system, β-blocking agents, calcium channel blockers, and diuretics.

### Efficacy

#### *Glycaemic parameters*

**Figure 1:**
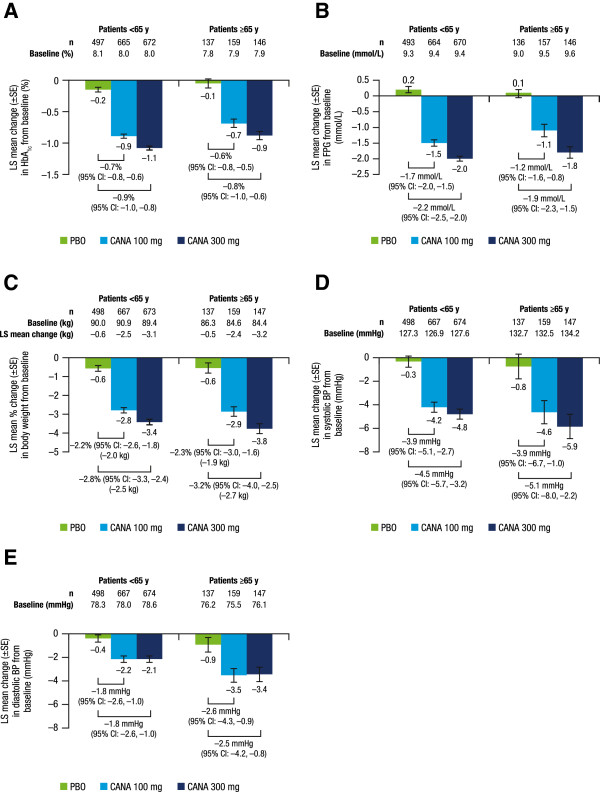
**Changes in efficacy parameters (LOCF). (A)** Change in HbA_1c_, **(B)** change in FPG, **(C)** percent change in body weight, **(D)** change in systolic BP, and **(E)** change in diastolic BP. LOCF, last observation carried forward; FPG, fasting plasma glucose; BP, blood pressure; LS, least squares; SE, standard error; CI, confidence interval; PBO, placebo; CANA, canagliflozin.

Canagliflozin 100 and 300 mg reduced HbA_1c_ compared with placebo in patients <65 and ≥65 years of age (Figure 1A). Placebo-subtracted HbA_1c_ reductions with canagliflozin 100 and 300 mg were -0.7% and -0.9%, respectively, in patients <65 years of age, and -0.6% and -0.8% in those ≥65 years of age. At Week 26, the proportion of patients <65 years of age achieving HbA_1c_ <7.0% was 45.6%, 60.0%, and 23.7% with canagliflozin 100 and 300 mg and placebo, respectively; 42.8%, 57.5%, and 28.5% of patients ≥65 years of age achieved HbA_1c_ <7.0% with canagliflozin 100 and 300 mg and placebo, respectively. Canagliflozin 100 and 300 mg also reduced FPG compared with placebo in both age subsets (Figure 1B). The effect of canagliflozin in lowering FPG was greater in patients <65 years of age (placebo-subtracted reductions for canagliflozin 100 and 300 mg of -1.7 and -2.2 mmol/L, respectively, in patients <65 years of age, and -1.2 and -1.9 mmol/L, respectively, in patients ≥65 years of age).

#### *Body weight and BP*

Canagliflozin 100 and 300 mg provided reductions in body weight in patients <65 and ≥65 years of age (Figure 1C), with differences versus placebo of -2.2% and -2.8%, respectively, in patients <65 years of age and -2.3% and -3.2%, respectively, in those ≥65 years of age. Both canagliflozin doses were associated with reductions in systolic and diastolic BP in both age subsets (Figures 1D and 1E). For systolic BP, placebo-subtracted reductions were -3.9 mmHg with canagliflozin 100 mg in both age subsets, and -4.5 and -5.1 mmHg with canagliflozin 300 mg in patients <65 and ≥65 years of age, respectively. Changes in diastolic BP with canagliflozin 100 and 300 mg were greater in patients ≥65 years of age (placebo-subtracted reductions of -2.6 and -2.5 mmHg, respectively) than in those <65 years of age (-1.8 mmHg for both doses). Mean changes in pulse rate with canagliflozin 100 and 300 mg and placebo were -0.9, -0.1, and 0.2 beats per minute (bpm), respectively, in patients <65 years of age and 0.6, -1.8, and -0.9 bpm, respectively, in those ≥65 years of age.

### Safety and tolerability

#### *Overall safety and tolerability*

The overall incidence of AEs was similar with canagliflozin and placebo within and across age subsets (Table 3). Incidences of serious AEs and AEs leading to discontinuation were low (≤3.3%) and similar across groups in patients <65 years of age. In patients ≥65 years of age, higher incidences of serious AEs and AE-related discontinuations were observed with canagliflozin 100 mg (6.9% and 8.8%, respectively) relative to canagliflozin 300 mg (3.4% and 5.4%, respectively) and placebo (3.6% and 4.4%, respectively); these increases with canagliflozin 100 mg were not associated with any pattern in specific AEs. Relative to placebo, both canagliflozin doses were associated with a higher incidence of AEs related to study drug in patients <65 and ≥65 years of age, which were mainly related to the specific AEs discussed below.

**Table 3:** Summary of overall safety and selected AEs^*^

	**Patients <65 y, n (%)**			**Patients ≥65 y, n (%)**		
	**PBO**	**CANA 100 mg**	**CANA 300 mg**	**PBO**	**CANA 100 mg**	**CANA 300 mg**
	**(n = 509)**	**(n = 674)**	**(n = 685)**	**(n = 137)**	**(n = 159)**	**(n = 149)**
Any AE	300 (58.9)	404 (59.9)	404 (59.0)	84 (61.3)	97 (61.0)	90 (60.4)
AEs leading to discontinuation	14 (2.8)	22 (3.3)	22 (3.2)	6 (4.4)	14 (8.8)	8 (5.4)
AEs related to study drug^†^	66 (13.0)	136 (20.2)	155 (22.6)	19 (13.9)	35 (22.0)	36 (24.2)
Serious AEs	17 (3.3)	17 (2.5)	17 (2.5)	5 (3.6)	11 (6.9)	5 (3.4)
Deaths	1 (0.2)	1 (0.1)	1 (0.1)	1 (0.7)	0	0
Selected AEs						
UTI	20 (3.9)	41 (6.1)	29 (4.2)	6 (4.4)	8 (5.0)	7 (4.7)
Genital mycotic infection						
Male^‡,§^	2 (0.8)	14 (4.3)	11 (3.4)	0	3 (3.7)	4 (5.2)
Female^||,¶^	10 (4.1)	37 (10.7)	44 (12.3)	0	7 (9.0)	5 (6.9)
Osmotic diuresis-related AEs^#^	4 (0.8)	44 (6.5)	39 (5.7)	1 (0.7)	12 (7.5)	8 (5.4)
Volume depletion-related AEs^**^	5 (1.0)	6 (0.9)	8 (1.2)	2 (1.5)	4 (2.5)	3 (2.0)
Renal-related AEs^††^	2 (0.4)	2 (0.3)	12 (1.8)	2 (1.5)	3 (1.9)	2 (1.3)

AE, adverse event; PBO, placebo; CANA, canagliflozin; UTI, urinary tract infection.

^*^All AEs are reported for regardless of rescue medication; hypoglycaemia episodes are reported for prior to rescue medication.

^†^Possibly, probably, or very likely related to study drug, as assessed by investigators.

^‡^For patients <65 years: PBO, n = 263; CANA 100 mg, n = 327; CANA 300 mg, n = 327; for patients ≥65 years: PBO, n = 71; CANA 100 mg, n = 81; CANA 300 mg, n = 77.

^§^Reported terms included balanitis, balanitis candida, balanoposthitis, and genital infection fungal in both age groups.

^||^For patients <65 years: PBO, n = 246; CANA 100 mg, n = 347; CANA 300 mg, n = 358; for patients ≥65 years: PBO, n = 66; CANA 100 mg, n = 78; CANA 300 mg, n = 72.

^¶^Reported terms included vaginal infection, vulvovaginal candidiasis, vulvovaginal mycotic infection, and vulvovaginitis in both age groups, and genital infection fungal and vulvitis in patients aged <65 years.

^#^Reported terms included micturition urgency, nocturia, pollakiuria, polyuria, dry mouth, polydipsia, and thirst in both age groups, and urine output increased in patients aged <65 years.

^**^Reported terms included dizziness postural, hypotension, and orthostatic hypotension in both age groups, and dehydration and syncope in patients aged <65 years.

^††^Reported terms included blood creatinine increased, glomerular filtration rate decreased, and renal impairment in both age groups, and renal failure acute in patients aged <65 years.

Canagliflozin 100 and 300 mg were associated with increased incidence of genital mycotic infections in males and females relative to placebo in patients in both age subsets. Incidence of genital mycotic infections was no greater in patients ≥65 years of age compared with those <65 years of age (Table 3). No serious genital mycotic infection AEs were reported and most were mild or moderate in intensity, as assessed by the investigator. Among female patients, 5 genital mycotic infection AEs led to study discontinuation in the <65 years subset (4 with canagliflozin 100 mg and 1 with canagliflozin 300 mg), and 1 led to study discontinuation in the ≥65 years subset (canagliflozin 300 mg). Two male canagliflozin-treated patients discontinued from the study due to genital mycotic infections in each age subset (both with canagliflozin 300 mg in the <65 years subset and both with canagliflozin 100 mg in the ≥65 years subset). The incidence of UTIs was higher with canagliflozin 100 mg relative to canagliflozin 300 mg and placebo in patients <65 years of age and similar across groups in those ≥65 years of age. Two serious UTIs were reported in patients <65 years of age (1 with canagliflozin 100 mg and 1 with canagliflozin 300 mg) and 1 UTI led to study discontinuation in the placebo group. No UTIs were serious or led to study discontinuation in patients ≥65 years of age.

A higher incidence of AEs related to osmotic diuresis (eg, pollakiuria [increased urination frequency], polyuria [increased urine volume]) was seen with canagliflozin relative to placebo in patients <65 and ≥65 years of age, with similar incidences observed in the 2 age subsets. Most AEs related to osmotic diuresis were mild or moderate in intensity, as assessed by the investigator, and none were serious; 3 patients experienced osmotic diuresis-related AEs that led to study discontinuation (1 with canagliflozin 100 mg and 2 with canagliflozin 300 mg), all in patients <65 years of age. The incidence of volume depletion-related AEs (eg, postural dizziness, orthostatic hypotension) was low (≤2.5%) across treatment groups in both age subsets. In patients <65 years of age, the incidence of volume depletion-related AEs was similar with canagliflozin 100 and 300 mg and placebo (0.9%, 1.2%, and 1.0%, respectively). Although there were few patients ≥65 years of age with volume depletion-related AEs, there was a trend toward a higher incidence with canagliflozin 100 and 300 mg relative to placebo (2.5%, 2.0%, and 1.5%, respectively). Most volume depletion-related AEs were mild or moderate in intensity, as assessed by the investigator. There was 1 serious volume depletion-related AE and 1 event that led to study discontinuation, both in the placebo group in 2 distinct patients <65 years of age.

A low incidence (<2%) of renal-related AEs (eg, glomerular filtration rate decreased, renal impairment) was seen in patients ≥65 years of age that was similar across treatment groups (1.9%, 1.3%, and 1.5% with canagliflozin 100 and 300 mg and placebo, respectively); a higher incidence was observed with canagliflozin 300 mg relative to canagliflozin 100 mg and placebo in patients <65 years of age (1.8%, 0.3% and 0.4%, respectively). Most renal-related AEs were mild or moderate in intensity, as assessed by the investigator, for patients <65 years of age, except for 1 serious renal-related AE with canagliflozin 100 mg. Overall, 10 patients discontinued due to renal-related AEs with canagliflozin, 6 patients <65 years of age (all with canagliflozin 300 mg), and 4 patients ≥65 years of age (3 with canagliflozin 100 mg and 1 with canagliflozin 300 mg); a renal-related AE led to study discontinuation in 1 patient ≥65 years of age with placebo.

Early transient decreases from baseline in eGFR were seen with canagliflozin within the first 3 to 6 weeks of treatment, which subsequently were stable or attenuated over the 26-week treatment period. At Week 26, canagliflozin 100 and 300 mg were associated with mean percent decreases in eGFR relative to placebo in patients <65 years of age (-1.6%, -3.0%, and -0.6%, respectively) and those ≥65 years of age (-2.6%, -2.9%, and -0.4%, respectively).

Among patients who were not on background sulphonylurea therapy (ie, excluding those from the add-on to metformin plus sulphonylurea study), the incidence of documented hypoglycaemia episodes with canagliflozin 100 and 300 mg and placebo was 4.1%, 4.0%, and 3.6%, respectively, in patients ≥65 years of age (n = 360); in those <65 years of age (n = 1,484), the incidence was 3.8%, 4.3%, and 1.8%, respectively. Canagliflozin was not associated with an increase in the incidence of severe hypoglycaemia episodes, with 2 events reported in patients <65 years of age (1 in each canagliflozin group) and none in patients ≥65 years of age. A total of 384 patients <65 years of age and 85 patients ≥65 years of age were on background sulphonylurea (ie, patients from the add-on to metformin plus sulphonylurea study); in these patients, the incidence of documented hypoglycaemia episodes with canagliflozin 100 and 300 mg and placebo was 27.8%, 26.1%, and 11.5%, respectively, in patients ≥65 years of age and 27.3%, 30.8%, and 16.2%, respectively, in patients <65 years of age. In patients on background sulphonylurea, there were no severe hypoglycaemia episodes reported in patients ≥65 years of age, and 2 severe hypoglycaemia episodes were reported in patients <65 years of age (1 with placebo and 1 with canagliflozin 100 mg).

#### *Fasting plasma lipids*

Overall, changes in lipid parameters with canagliflozin compared with placebo were generally similar in patients <65 and ≥65 years of age (Table 4). Increases in HDL-C were seen with canagliflozin 100 and 300 mg relative to placebo in both age subsets (differences vs placebo of 5.2% and 6.1%, respectively, in patients <65 years of age, and 6.2% and 7.2%, respectively, in patients ≥65 years of age). Canagliflozin 100 and 300 mg were associated with increases in LDL-C relative to placebo in both age subsets (differences vs placebo of 4.8% and 8.3%, respectively, in patients <65 years of age, and 2.7% and 6.1%, respectively, in patients ≥65 years of age), with increases in non–HDL-C that were smaller than those seen in LDL-C. Relative to placebo, there were no meaningful changes in triglycerides or the LDL-C/HDL-C ratio with canagliflozin in both age subsets.

**Table 4:** Summary of changes from baseline in fasting plasma lipids at Week 26 (LOCF)^*^

	**Patients <65 y**			**Patients ≥65 y**		
	**PBO**	**CANA 100 mg**	**CANA 300 mg**	**PBO**	**CANA 100 mg**	**CANA 300 mg**
Triglycerides, n	446	614	610	118	139	131
Mean ± SD baseline, mmol/L	2.2 ± 1.4	2.1 ± 1.5	2.1 ± 1.5	1.8 ± 0.8	1.9 ± 1.1	1.7 ± 0.9
LS mean ± SE change	0.01 ± 0.05	-0.10 ± 0.04	-0.23 ± 0.04	-0.05 ± 0.05	-0.14 ± 0.05	-0.16 ± 0.05
Median (IQR) percent change	-1.6	-6.0	-9.3	-3.2	-7.4	-9.2
	(-22.1, 28.8)	(-26.4, 24.4)	(-28.6, 19.8)	(-20.4, 16.3)	(-21.9, 10.6)	(-25.8, 10.2)
LS mean ± SE percent change	9.1 ± 2.2	3.7 ± 2.0	0.6 ± 2.0	2.6 ± 3.0	-2.4 ± 2.8	-2.5 ± 2.9
Difference versus PBO (95% CI)		-5.4 (-11.1, 0.3)	-8.5 (-14.2, -2.8)		-5.0 (-13.0, 3.1)	-5.1 (-13.3, 3.1)
LDL-C, n	444	609	601	118	137	129
Mean ± SD baseline, mmol/L	2.9 ± 1.0	2.8 ± 0.9	2.7 ± 0.9	2.7 ± 1.0	2.6 ± 0.9	2.7 ± 1.0
LS mean ± SE change	-0.05 ± 0.03	0.06 ± 0.03	0.17 ± 0.03	-0.07 ± 0.06	0.04 ± 0.05	0.07 ± 0.06
Median (IQR) percent change	-2.3	2.0	6.5	-1.5	1.9	2.0
	(-16.9, 11.3)	(-10.7, 19.4)	(-7.8, 23.9)	(-9.8, 12.2)	(-7.1, 15.5)	(-10.5, 17.9)
LS mean ± SE percent change	1.5 ± 1.4	6.3 ± 1.2	9.8 ± 1.2	0.7 ± 2.1	3.5 ± 1.9	6.8 ± 2.0
Difference versus PBO (95% CI)		4.8 (1.2, 8.4)	8.3 (4.7, 11.9)		2.7 (-2.9, 8.4)	6.1 (0.4, 11.8)
HDL-C, n	446	612	606	118	138	129
Mean ± SD baseline, mmol/L	1.2 ± 0.3	1.2 ± 0.3	1.2 ± 0.3	1.2 ± 0.3	1.3 ± 0.3	1.3 ± 0.3
LS mean ± SE change	0.03 ± 0.01	0.09 ± 0.01	0.10 ± 0.01	0.03 ± 0.02	0.12 ± 0.02	0.12 ± 0.02
Median (IQR) percent change	3.2	7.3	9.5	3.9	9.2	9.7
	(-6.0, 13.6)	(-2.6, 19.6)	(-0.7, 20.0)	(-4.7, 11.8)	(0.0, 17.6)	(-0.8, 21.2)
LS mean ± SE percent change	4.0 ± 0.8	9.2 ± 0.7	10.2 ± 0.7	3.6 ± 1.4	9.8 ± 1.3	10.8 ± 1.4
Difference versus PBO (95% CI)		5.2 (3.1, 7.3)	6.1 (4.1, 8.2)		6.2 (2.4, 9.9)	7.2 (3.4, 11.0)
LDL-C/HDL-C, n	444	609	601	118	137	129
Mean ± SD baseline, mol/mol	2.6 ± 1.1	2.5 ± 1.0	2.4 ± 0.9	2.3 ± 1.0	2.2 ± 0.9	2.2 ± 0.8
LS mean ± SE change	-0.13 ± 0.03	-0.12 ± 0.03	-0.07 ± 0.03	-0.10 ± 0.05	-0.15 ± 0.05	-0.11 ± 0.05
Median (IQR) percent change	-6.8	-5.4	-1.5	-4.4	-3.3	-7.3
	(-19.3, 9.3)	(-19.0, 10.9)	(-16.3, 14.1)	(-19.8, 12.8)	(-17.4, 7.8)	(-21.9, 11.8)
LS mean ± SE percent change	-0.5 ± 1.4	-0.7 ± 1.2	1.3 ± 1.2	-1.4 ± 2.3	-4.2 ± 2.2	-1.2 ± 2.3
Difference versus PBO (95% CI)		-0.2 (-3.8, 3.4)	1.8 (-1.8, 5.4)		-2.8 (-9.1, 3.4)	0.2 (-6.2, 6.5)
Non–HDL-C, n	446	609	602	117	138	127
Mean ± SD baseline, mmol/L	3.9 ± 1.1	3.8 ± 1.1	3.6 ± 1.0	3.5 ± 1.2	3.5 ± 1.1	3.5 ± 1.1
LS mean ± SE change	-0.04 ± 0.04	0.00 ± 0.03	0.10 ± 0.03	-0.09 ± 0.07	-0.02 ± 0.06	-0.02 ± 0.06
Median (IQR) percent change	-2.3	-0.7	2.4	-2.6	0.0	0.0
	(-13.6, 9.7)	(-10.2, 13.1)	(-8.6, 14.9)	(-10.6, 9.5)	(-6.3, 11.5)	(-12.6, 13.5)
LS mean ± SE percent change	1.1 ± 1.0	2.6 ± 0.9	4.7 ± 0.9	-0.5 ± 1.8	0.8 ± 1.7	2.8 ± 1.8
Difference versus PBO (95% CI)		1.4 (-1.3, 4.1)	3.6 (0.8, 6.3)		1.3 (-3.6, 6.2)	3.3 (-1.7, 8.3)

LOCF, last observation carried forward; PBO, placebo; CANA, canagliflozin; SD, standard deviation; LS, least squares; SE, standard error; IQR, interquartile range; CI, confidence interval; LDL-C, low-density lipoprotein cholesterol; HDL-C, high-density lipoprotein cholesterol.

^*^All fasting plasma lipids parameters are reported for regardless of rescue medication.

## Discussion

The treatment of older patients with T2DM must take into consideration factors specific to these patients, including the increased prevalence of comorbidities and use of concomitant medications, with patient safety being a priority [1,19]. For example, safety issues related to available AHAs that may be of particular concern in older patients include gastrointestinal intolerability and contraindication in patients with renal impairment for metformin; risk of congestive heart failure and fracture with thiazolidinediones; and risk of hypoglycaemia with sulphonylureas and insulin [5]. In this analysis, the efficacy and safety of canagliflozin were evaluated using pooled data from patients with T2DM <65 and ≥65 years of age. Canagliflozin 100 and 300 mg improved glycaemic control relative to placebo in patients <65 and ≥65 years of age, with a slightly greater effect observed in patients aged <65 years relative to those aged ≥65 years. Canagliflozin provided reductions in body weight and systolic and diastolic BP compared with placebo in both age subsets. Canagliflozin was generally well tolerated, with higher incidences relative to placebo of genital mycotic infections and osmotic diuresis-related AEs in both age subsets. Findings in this analysis were consistent with those from previous studies, including one in older patients ≥55 to ≤80 years of age [9-16].

A slightly numerically lesser effect of canagliflozin in lowering HbA_1c_ and FPG was observed in patients ≥65 years of age relative to those <65 years of age, which may be related to the lower mean baseline HbA_1c_ and eGFR in older patients. The mechanism of action of canagliflozin is through induction of UGE, the rate of which is dependent on plasma glucose concentration and GFR [20-22]; thus, the effect of canagliflozin in increasing UGE is expected to be attenuated in patients with lower eGFR. For example, canagliflozin improved glycaemic control to a lesser extent in patients with moderate renal impairment (eGFR ≥30 and <50 mL/min/1.73 m^2 ^[13]) relative to patients with normal or mildly impaired renal function [9,10,12]. Consistent with this, reductions in HbA_1c_ and FPG with canagliflozin in the previous study in older patients [11] were smaller than those seen in other studies with generally younger patients [9,10,12,14-16].

Body weight was reduced with both canagliflozin doses relative to placebo in patients <65 and ≥65 years of age, with generally similar effects in the 2 age subsets. Canagliflozin was associated with greater reductions from baseline in systolic and diastolic BP relative to placebo in both age subsets; reductions in diastolic BP were numerically larger in patients ≥65 years versus <65 years of age. The reductions in BP with canagliflozin were not associated with notable changes in pulse rate or incidence of AEs related to volume depletion in either age group.

Canagliflozin 100 and 300 mg were generally well tolerated in patients <65 and ≥65 years of age. The incidence of overall AEs was similar across treatment groups in both age subsets, with no notable increase in patients ≥65 years relative to those <65 years of age. Serious AEs and AEs leading to study discontinuation were slightly more common with canagliflozin 100 mg relative to canagliflozin 300 mg and placebo in patients ≥65 years of age, with numerical differences not associated with any pattern in specific AEs. As previously observed [9-13], canagliflozin was associated with increased incidences of genital mycotic infections and osmotic diuresis-related AEs, with no notable differences observed between the 2 age subsets. The incidence of volume depletion-related AEs was low and similar with canagliflozin and placebo in patients <65 years of age; the incidence of these AEs was generally higher in patients ≥65 years of age, with slightly higher rates with canagliflozin relative to placebo. In patients <65 years of age, the incidence of renal-related AEs was low but higher with canagliflozin 300 mg relative to canagliflozin 100 mg and placebo, while the incidence of these AEs was low and similar across groups in patients ≥65 years of age. Canagliflozin was not associated with increased incidences of UTIs or documented or serious hypoglycaemia episodes in patients ≥65 years of age. The relatively low risk of hypoglycaemia observed with canagliflozin when not used in combination with agents associated with hypoglycaemia (eg, insulin and sulphonylurea) is consistent with its mechanism of action, as canagliflozin lowers the RT_G_ to approximately 4.4 to 5.0 mmol/L [8,17], above the usual threshold for hypoglycaemia of 3.9 mmol/L. In this analysis, the number of patients on background AHAs associated with hypoglycaemia was small; in these patients, an increased incidence of documented hypoglycaemia was observed with canagliflozin in both age subsets, with no increase in severe events, consistent with previous studies of canagliflozin in patients on background insulin or sulphonylurea [11,13,15,23]. Overall, the safety profile of canagliflozin relative to placebo in this analysis was consistent with that seen in previous studies, including those in generally younger patient populations [9,11,13-16]. There was no consistent dose-related increase in the incidence of several AEs (eg, UTIs, genital mycotic infections, osmotic diuresis-related AEs) in patients <65 or ≥65 years of age.

Both canagliflozin doses were associated with increases in HDL-C and LDL-C, and no change in the LDL-C/HDL-C ratio, relative to placebo. The changes in lipid parameters with canagliflozin were generally similar in patients <65 and ≥65 years of age, and were consistent with previous observations in individual Phase 3 studies [9-16]. Similar changes in lipid parameters have been seen with other SGLT2 inhibitors [24,25]. The mechanism for the increase in LDL-C seen with canagliflozin is unknown. In previous studies in which changes in apolipoprotein B (Apo B) were assessed, canagliflozin was associated with increases in Apo B that were smaller than those in LDL-C [9,14].

Findings from this integrated analysis extend those from a previous study in older patients ≥55 to ≤80 years of age [11] by providing a direct comparison of the efficacy and safety of canagliflozin in patients <65 and ≥65 years of age based on a larger, pooled patient population representing a broad and general population of patients with T2DM. A limitation of this analysis was the lack of pre-specified statistical testing for comparisons between the age groups; in addition, the Phase 3 studies pooled in this report were not powered to evaluate statistical differences between age subgroups. However, 95% CIs for the comparisons of canagliflozin 100 and 300 mg relative to placebo within each age subset are reported. Assessments over a longer treatment period and including active comparators will be helpful in further defining the efficacy/safety profile of canagliflozin relative to other available AHAs as treatment options for older patients with T2DM. Other issues that may impact the management of T2DM, such as self-management ability, cognitive function, need for carer support, and frailty [4,5], will also be important considerations in the selection of AHA treatment for older patients with T2DM.

## Conclusions

Improvements in glycaemic control, body weight, and systolic BP seen with canagliflozin were generally consistent between younger (<65 years of age) and older (≥65 years of age) patients. Moreover, there were no notable differences in the safety and tolerability profile of canagliflozin between these age subsets. Thus, these findings suggest that canagliflozin is an effective and generally well tolerated treatment option for older patients with T2DM.

## Abbreviations

AE: Adverse event; AHA: Antihyperglycaemic agent; ALT: Alanine aminotransferase; ANCOVA: Analysis of covariance; Apo B: Apolipoprotein B; BMI: Body mass index; BP: Blood pressure; bpm: beats per minute; CANA: Canagliflozin; CI: Confidence interval; CV: Cardiovascular; eGFR: estimated glomerular filtration rate; FPG: Fasting plasma glucose; HDL-C: High-density lipoprotein cholesterol; IQR: Interquartile range; LDL-C: Low-density lipoprotein cholesterol; LOCF: Last observation carried forward; LS: Least squares; MedDRA: Medical Dictionary for Regulatory Activities; MET: Metformin; mITT: Modified intent-to-treat; PBO: Placebo; PIO: Pioglitazone; RTG: Renal threshold for glucose; SD: Standard deviation; SE: Standard error; SGLT2: Sodium glucose co-transporter 2; SU: Sulphonylurea; T2DM: Type 2 diabetes mellitus; UGE: Urinary glucose excretion; ULN: Upper limit of normal; UTI: Urinary tract infection.

## Competing interests

AS has received lecture and/or advisory board fees from Takeda, Novartis, Eli Lilly and Company, and Merck Sharp & Dohme. BB has served as investigator using research and grant support received by his institution from Janssen Research & Development, LLC. SH has served on advisory boards for Sanofi-Aventis, Janssen, Novo Nordisk, Takeda, AstraZeneca, Bristol-Myers Squibb, Eli Lilly and Company, Boehringer Ingelheim; served as a speaker for Sanofi-Aventis, AstraZeneca, Bristol-Myers Squibb, Eli Lilly and Company, Boehringer Ingelheim, and Novo Nordisk; and has received research support from Sanofi-Aventis, Novo Nordisk, and Merck. UV, CM, AF, WS, KU, MD, and GM are current or former full-time employees of Janssen Research & Development, LLC. CM is a holder of Johnson & Johnson stock and stock options.

## Authors’ contributions

AS, BB, and SH contributed to the interpretation of data, and drafted, reviewed, and approved the manuscript. AF, WS, KU, MD, and GM contributed to the design and conduct of the analysis; the acquisition, analysis, and interpretation of data; and drafted, reviewed, and approved the manuscript. UV and CM contributed to the analysis and interpretation of the data, and drafted, reviewed, and approved the manuscript. All authors read and approved the final manuscript.

## Pre-publication history

The pre-publication history for this paper can be accessed here:

http://www.biomedcentral.com/1472-6823/14/37/prepub

## Supplementary Material

Additional file 1 List of institutional review boards (IRBs) and independent ethics committees (IECs) by study. Click here for file
