# Supplementary material for: Efficacy and safety of canagliflozin compared with placebo in older patients with type 2 diabetes mellitus: a pooled analysis of clinical studies
Source: BMC Endocr Disord. 2014 Apr 18;14:37. doi: 10.1186/1472-6823-14-37 (PMC4021426; doi:10.1186/1472-6823-14-37)
Supplement: Additional file 1 — List of institutional review boards (IRBs) and independent ethics committees (IECs) by study. [file 1472-6823-14-37-S1.docx]

### Additional file 1 – List of institutional review boards (IRBs) and independent ethics committees (IECs) by study

|  |  |  | **Monotherapy** | **Add-on to**  **MET** | **Add-on to**  **MET + SU** | **Add-on to**  **MET + PIO** |
| --- | --- | --- | --- | --- | --- | --- |
| **IRB/IEC** | **City** | **Country** |  |  |  |  |
| CODEI | Buenos Aires | Argentina |  | x |  |  |
| Comite de Docencia e Investigacion de CER San Juan | San Juan | Argentina |  | x |  |  |
| Comite de Docencia e Investigacion CEDIC | Buenos Aires | Argentina |  | x |  |  |
| Comite de Docencia e Investigacion IDIM | Buenos Aires | Argentina |  | x |  |  |
| Comité de Revisión de Actividades de Investigación - CRAI | Mendoza | Argentina |  | x |  |  |
| Comité de Revisión de Protocolos CIPREC | Buenos Aires | Argentina |  | x |  |  |
| Comite de Revision Institucional de los Cons Asoc de Endocrinologia e Invest Clin Aplicada | Buenos Aires | Argentina |  | x |  |  |
| Comite Independiente de Etica para Ensayos en Farmacologia Clinica | Buenos Aires | Argentina |  | x |  |  |
| Austin Health Human Research Ethics Committee | Heidelberg, Victoria | Australia |  |  | x |  |
| Metro South Health Service District Human Research Ethics Committee, Princess Alexandra Hospital | Woolloongabba | Australia |  |  | x |  |
| Sir Charles Gairdner Group Human Research Ethics Committee | Nedlands | Australia |  |  | x |  |
| South Metropolitan Area Health Service HREC | Fremantle | Australia |  |  | x |  |
| The University of Wollongong & Sesiahs Health & Medical Human Research Ethics Committee | Wollongong | Australia |  |  | x |  |
| Ethikkommission der Med. Fakultät der Univ. Wien un des AKH der Stadt Wien | Wien | Austria | x |  |  |  |
| Ethikkommission der Stadt Wien Gesundheitswesen und Soziales | Wien | Austria | x |  |  |  |
| Ethikkommission für das Bundesland Salzburg | Salzburg | Austria | x |  |  |  |
| Ethikkommission Für das Land NÖ am Sitz des Amtes der NÖ | St. Pölten | Austria | x |  |  |  |
| Commissie Medische Ethiek UZ Leuven | Leuven | Belgium |  |  | x |  |
| Commissie Medische Ethiek UZA | Edegem | Belgium |  |  | x |  |
| Imelda VZW Commissie Medische Ethiek | Bonheiden Antwerpen | Belgium |  |  | x |  |
| O.L.V. Ziekenhuis | Aalst | Belgium |  |  | x |  |
| Ethics Committee for Multicenter Trials | Sofia | Bulgaria |  | x |  |  |
| Ethics Committee of DCC Akta Medica Sevlievo | Sofia | Bulgaria |  | x |  |  |
| Ethics Committee of Tokuda Hospital Sofia | Sofia | Bulgaria |  | x |  |  |
| Ethics Committee of UMHAT "Dr Georgi Stranski" Pleven | Pleven | Bulgaria |  | x |  |  |
| Ethics Committee of UMHAT "St George" Plovdiv | Plovdiv | Bulgaria |  | x |  |  |
| Research Ethics Review Committee (RERC) | Edmonton | Canada |  |  |  | x |
| Trafalgar Ethics Board | Oakville | Canada |  |  |  | x |
| Comite de Etica de Investigacion de Riesgo de Fractura | Bogota | Colombia |  | x |  |  |
| Comité de Ética en Investigacion de la Clinica de la Costa | Barranquilla | Colombia | x | x |  |  |
| Comité de Ética en la Invesstigación CAIMED | Bogota | Colombia | x | x |  |  |
| Comite de Investigaciones y Etica en Investigacion - HPTU | Medellin | Colombia |  | x |  |  |
| Comite Institucional de Etica e Investigacion Clinica (CIEIC) | Bogota | Colombia | x | x |  |  |
| Eticka komise IKEM a FTNsP | Praha | Czech Republic |  | x |  |  |
| Tallinn Medical Research Ethics Committee | Tallinn | Estonia | x | x |  |  |
| Pohjois-Savon shpn eettinen toimikunta | Kuopio | Finland |  |  |  | x |
| CPP Ile de France VI | Paris | France |  |  | x |  |
| CPP Ile de France X | Aulnay-sous-Bois | France |  |  |  | x |
| Ethik Kommission bei der Bayerischen Landesärztekammer | München | Germany |  |  |  | x |
| Ethikkommission bei der Saechsischen Landesärztekammer | Dresden | Germany |  |  |  | x |
| Ethikkommission der Landesärztekammer Rheinland-Pfalz | Mainz | Germany |  |  |  | x |
| Leicestershire, Northamptonshire & Rutland Research Ethics Committee 1 | Nottingham | Great Britain |  |  | x | x |
| Leicestershire, Northamptonshire & Rutland Research Ethics Committee 2 | Nottingham | Great Britain |  |  | x | x |
| National Ethics Committee | Athens | Greece |  | x |  | x |
| Comite de Etica Independiente Zugueme | Col. Tecun Uman | Guatemala | x |  | x |  |
| Medical Research Council Ethics Committee for Clinical Pharmacology | Budapest | Hungary |  |  | x |  |
| The National Bioethics Committee | Reykjavik | Iceland | x |  |  |  |
| Bangalore Central Ethics Committee | Bangalore | India | x |  |  |  |
| Central India Medical Research Ethics Committee | Ramdaspeth, Nagpur | India | x |  |  |  |
| Clinicom | Bangalore | India | x | x |  | x |
| Ethical Control Committee | Aurangabad Maharashtra | India |  | x |  |  |
| Ethics Committee of Diabetes Care n Research Centre | Nagpur Maharashtra | India |  |  |  | x |
| Global Health Concern Ethics Committee | Nagpur Maharashtra | India |  | x |  |  |
| Gujarat Kidney Foundation Ethical Committee | Ahmedabad | India |  | x |  |  |
| HCG Medisurge Ethics Committee | Ahmedabad Gujarat | India |  |  |  | x |
| Independent Human Ethics Committee, Health and Research Centre | Trivandrum Kerala | India |  | x |  |  |
| Institutional Ethics Committee of Kovai Diabetes Speciality Centre and Hospital | Coimbatore | India |  | x |  |  |
| KGN Ethics Committee | Mumbai Maharashtra | India |  | x |  |  |
| National Ethics Committee | Chennai Tamil Nadu | India |  |  |  | x |
| Regional Ethics Committee | Coimbatore Tamil Nadu | India |  |  |  | x |
| Sahyadri Ethics Committee | Pune | India |  | x |  |  |
| Science for Health | Bangalore | India |  | x |  |  |
| St. John's Medical College and Hospital Ethics Committee | Bangalore | India |  | x |  |  |
| Swasthya Kalyan Ethics Committee | Jaipur Rajasthan | India |  |  |  | x |
| Helsinki Committee - Hadassah MC | Jerusalem | Israel |  |  | x |  |
| Helsinki Committee - Meir MC | Kfar Saba | Israel |  |  | x |  |
| Helsinki Committee - Rambam MC | Haifa | Israel |  |  | x |  |
| Helsinki Committee - Sourasky Medical Center | Tel Aviv | Israel |  |  | x |  |
| Helsinki Committee - Wolfson MC | Holon | Israel |  |  | x |  |
| Comitato di Etica per la Ricerca Biomedica Dell'Universitá Degli Studi Gabriele D'Annuzio e Asl | Chieti | Italy |  | x |  |  |
| Comitato Etico Dell'A.O.U. di Cagliari Dâ'Aloja | Cagliari | Italy |  |  |  | x |
| Comitato Etico Dell'Azienda Ospedaliera Universitaria Mater Domini di Catanzaro Ricci | Catanzaro | Italy |  | x |  | x |
| Comitato Etico Dell'IRCCS - Fondazione San Raffaele Del Monte | Milano | Italy |  | x |  |  |
| Comitato Etico Indipendente Presso La Fondazion Ptv Policlinico Tor Vergata di Roma | Roma | Italy |  | x |  |  |
| Chonbuk National University Hospital | Jeonju-Si | Korea | x |  |  |  |
| Dongguk University Ilsan Hospital | Goyangsi Gyeonggido | Korea | x |  |  |  |
| IRB of Busan Paik Hospital Inje University | Busan | Korea | x |  |  |  |
| IRB of Kangbuk Samsung Hospital | Seoul | Korea | x |  |  |  |
| IRB of Yonsei University Wonju College of Medicine, Wonju Christian Hospital | Wonju | Korea | x |  |  |  |
| EC for Clinical Research at P Stadins CUH Development Society | Riga | Latvia |  | x |  |  |
| Lithuanian Bioethics Committee | Vilnius | Lithuania | x |  |  |  |
| Jawatankuasa Etika Penyelidikan (Manusia) USM | Kubang Kerian kelantan | Malaysia | x | x |  |  |
| Medical Research and Ethics Committee (MREC) | Kuala Lumpur | Malaysia |  | x |  |  |
| Medical Ethics Committee, University Malaya Medical Centre | Kuala Lumpur | Malaysia | x | x |  |  |
| Sekretariat Penyelidikan Perubatan & Industri, Pusat Perubatan Universiti | Cheras Selangor | Malaysia | x | x |  |  |
| Comision de Etica e Investigacion | Col. Los Angeles Durango | Mexico |  |  |  | x |
| Comité de Bioetica del Centro Universitario de Ciencias de la Salud | Guadalajara | Mexico | x |  |  |  |
| Comité de Bioética del Departmento de Fisiología del Centro Universitario de Ciencias de la Salud | Guadalajara | Mexico |  | x |  |  |
| Comite de Bioetica en Investigacion del Instituto de Corazon de Queretaro | Queretaro | Mexico |  | x |  |  |
| Comité de Ética Central Médico Quirurgica de Aguascalientes | Aguascalientes | Mexico | x |  |  |  |
| Comité de Ética de Investigación - Cardiolink Clin Trials | Monterrey | Mexico | x |  |  |  |
| Comite de Etica de la Clinica Roma | Monterrey | Mexico | x |  | x |  |
| Comite de Etica de la Facultad de Medicina de la UANL y Hospital Universitario | Monterrey | Mexico |  | x | x |  |
| Comité de Ética e Investigación del Noreste S C | Naranjal Tampico, Tamaulipas | Mexico |  | x |  |  |
| Comite de Etica Independiente en Investigacion Cientifica | Monterrey | Mexico |  |  | x |  |
| Comité de Ética Instituto Jalisciense de Investigación Clinica S.A. de C.V. | Guadalajara | Mexico | x |  | x |  |
| Comite de Eticauis | Col. San Felipe Chihuahua | Mexico |  |  |  | x |
| Comité de Investigación Para Estudios en Humanos | Delegación Tlalpan, Mexico City | Mexico | x |  |  |  |
| Comite de Investigacion Paracelsus | Col. San Rafael Cuauhtemoc | Mexico |  |  |  | x |
| Comité de Revisión Interna y Ética en Investigación de Sociedad Médica del Hospital Ángeles Culiacan | Culiacan, Sinaloa | Mexico |  | x |  |  |
| Instituto Nacional de Ciencias Medicas y Nutrición Salvador Zubirán | Mexico City | Mexico |  |  | x |  |
| Comisión Reguladora de Estudios Clínicos del Complejo Hospitalario San Pablo | Lima | Peru |  | x |  |  |
| Comité de Etica del Hospital Nacional Edgardo Rebagliatti Martins | Lima | Peru |  | x |  |  |
| Comite de Etica en Investigacion Biomedica del Hospital Nacional Cayetano Heredia | Lima | Peru |  | x |  |  |
| Comite de Etica en Investigacion Biomedica del Hospital Nacional Dos de Mayo | Lima | Peru |  | x |  |  |
| Comite Instituciónal de Etica en Investigación Universidad San Martin de Porres Clínica Cada Mujer | Lima | Peru |  | x |  |  |
| Ethics Review Committee, Ospital ng Makati | Makati City | Philippines | x |  |  |  |
| Hospital Ethics Review Committee, San Juan de Dios Hospital | Pasay City | Philippines | x |  |  |  |
| Technical and Ethical Review Board, Manila Doctors Hospital | Ermita, Manila | Philippines | x |  |  |  |
| UERMMMC-ISDFI Ethics Committee | Marikina City | Philippines | x |  |  |  |
| Komisja Bioetyczna Kujawsko-Pomorska Okręgowa Izba Lekarska w Toruniu | Toruń | Poland | x |  |  |  |
| Komisja Bioetyczna przy OIL w Lublinie | Lublin | Poland |  | x |  |  |
| Comissao de Etica para a Investigacao Clinica | Lisboa | Portugal |  | x |  |  |
| Academia de Stiinte Medicale Comisia Nationala de Etica Pentru Studiul Medicamentului | Bucuresti | Romania | x |  |  |  |
| Ethic Committee of FSI Federal Centre of Heart, Blood, and Endocrinology na VA Almazov Rosmedtekhn | St. Petersburg | Russia |  | x |  |  |
| Ethic Committee of Outpatient Clinic #3 of President's Medical Centre | Moscow | Russia |  | x |  |  |
| Ethics Committee of Diagnostic Center #85 | St. Petersburg | Russia |  | x |  |  |
| Independent Interdisciplinary Committee for Ethics Expertise of Clinical Trials | Moscow | Russia |  | x | x |  |
| Centralized Independent Review Board | Singapore | Singapore |  | x |  |  |
| Domain Specific Review Board | Singapore | Singapore |  | x |  |  |
| Eticka komisia KSK | Košice | Slovakia |  | x |  |  |
| MEC - Etická komisia BSK | Bratislava | Slovakia |  | x |  |  |
| Etická komisia NSK | Nitra | Slovakia |  | x |  |  |
| Nezávislá etická komisia Banskobystrického samosprávneho kraja | Banská Bystrica | Slovakia |  | x |  |  |
| Pharma Ethics | Pretoria | South Africa | x |  |  |  |
| CEIC Área 3 - Hospital Universitario Principe de Asturias de Alcalá de Henares | Alcalá de Henares | Spain | x |  |  |  |
| CEIC Área 6 - Hospital Universitario Puerta de hierro de majadahonda | Madrid | Spain | x |  |  |  |
| CEIC Autonómico de Ensayos Clínicos de Andalucía | Sevilla | Spain |  |  | x | x |
| CEIC Clinica Mediterránea de Neurociencias | Partida de Bacarot | Spain |  |  | x |  |
| CEIC Hospital Clinico Universitario de Valencia | Valencia | Spain |  |  | x |  |
| CEIC Hospital Clinico Universitario Virgen de la Victoria | Malága | Spain |  |  | x |  |
| CEIC Hospital General Universitario de Elche | Elche | Spain | x |  |  |  |
| CEIC Hospital Torrecárdenas | Alméria | Spain |  |  | x | x |
| CEIC Hospital Universitari de Girona | Girona | Spain | x |  |  |  |
| CEIC Hospital Virgen del Rocío | Sevilla | Spain |  |  |  | x |
| Comite Etico de Ensayos Clinicos Hospital Ramon y Cajal | Madrid | Spain |  |  |  | x |
| EC of Hospital Univer. Virgen Macarena | Sevilla | Spain |  |  | x |  |
| Regionala etikprövningsnämnden i Göteborg | Göteborg | Sweden | x | x |  |  |
| Ethical Clearance Committee on Human Rights Related to Researches Involving Human Subjects, Ramathibodi Hospital | Bangkok | Thailand |  | x |  | x |
| Ethics Committees on Researches Involving Human Subjects, Rajavithi Hospital | Bangkok | Thailand |  | x |  | x |
| Institute for the Development of Human Research Protections | Nonthaburi | Thailand |  | x |  | x |
| Institutional Review Board Sueblingvong, Tada, IRB/IEC | Bangkok | Thailand |  | x |  |  |
| Institutional Review Board, Royal Thai Army Medical Department | Bangkok | Thailand |  |  |  | x |
| Khon Kaen University Ethics Committee for Human Research | Khon Kaen | Thailand |  | x |  | x |
| Research Ethics Committee | Chiang Mai | Thailand |  | x |  |  |
| Istanbul University, Istanbul Medical Faculty Clinical Research Ethics Committee | Istanbul | Turkey |  | x |  |  |
| Central Ethics Commission of MoH of Ukraine | Kiev | Ukraine |  | x |  |  |
| Baylor Research Institute Institutional Review Board | Dallas, TX | United States |  |  | x |  |
| Human Subjects Protection Program, The University of Arizona | Tuscon, AZ | United States |  |  |  | x |
| LSU Health Science Center | New Orleans, LA | United States |  |  | x |  |
| Meharry Institutional Review Board | Nashville, TN | United States |  | x |  | x |
| Mercy Medical Center - Des Moines Institutional Review Committee | Des Moines, IA | United States |  |  |  | x |
| St. Luke's-Roosevelt Hospital Center Institute for Health Sciences IRB | New York, NY | United States | x |  |  |  |
| Sterling Institutional Review Board | Atlanta, GA | United States | x | x | x | x |
| University & Medical Center IRB, East Carolina University | Greenville, NC | United States |  |  | x |  |
| University of Vermont Research Protections Office | Colchester, VT | United States |  |  | x |  |
| Western Institutional Review Board | Olympia, WA | United States |  |  |  | x |

IRB, institutional review board; IEC, independent ethics committee; MET, metformin; SU, sulphonylurea; PIO, pioglitazone.
